# Supplementary material for: Who benefits from the donor-supported malaria programme in Enugu State, Nigeria? A benefit incidence analysis
Source: PLOS Glob Public Health. 2025 Mar 13;5(3):e0004286. doi: 10.1371/journal.pgph.0004286 (PMC11906064; doi:10.1371/journal.pgph.0004286)
Supplement: Attachment — Submitted filename: Response to Reviewer.docx 22072024wm.docx August.docx [file pgph.0004286.s002.docx]

**Response to Reviewer’s Comments**

5. Review Comments to the Author

Please use the space provided to explain your answers to the questions above. You may also include additional comments for the author, including concerns about dual publication, research ethics, or publication ethics. (Please upload your review as an attachment if it exceeds 20,000 characters)

Reviewer #1: I enjoyed reading this manuscript and I believe it addresses an important knowledge gap in malaria financing and control especially for Nigeria. I have few suggestions for the authors to consider.

1. I think the paper would benefit more with additional editing to make the sentences more succinct and clearer. For instance:

(a) Page 4 line 94-98 is a very long winding sentence that could be broken into two or three sentences for clarity.

**Response:** Thank you, we have modified this sentence (with track changes on page 4, line 93 to 99) and polished the language of the whole manuscript.

(b) Page 6, line 137 "..based on the operational of donor funded malaria programs" Should this read as "based on the operations of donor funded malaria program?" The word "operational' seem like is being misused here.. please check and confirm.

**Response:** This has been corrected to read “based on the operations of donor funded malaria program” on page 6, line 138.

(c) Page 10 line 231, I believe it was meant to read "SES of respondents in study sites" and not "sties"

**Response:** This has been corrected on page 10, line 237 to read "SES of respondents in study sites"

There are few other areas that could benefit more with careful editorial review by the authors.

Response: Thank you and we have polished the whole manuscript.

2. Given the broad definition of Benefits studies in financing and Economics, I suggest the authors describe clearly what type of benefits they are focusing on. To some readers, they may be thinking of benefits accrued from productivity work days avoided by not falling sick from malaria. I think it would be beneficial for authors to be clear upfront on how they intend to measure these benefits and why such approach is appropriate in this context.

**Response:** Thank you for this highlight. We have explained in track changes with references on page 7, line 172 to 174 the type of benefits that we focused on.

3. Authors use words that are not defined or could be unclear to authors. For example on Page 10 the last sentence, line 247, '... each study groups were either in big business or..." It is unclear to the reader what 'big business' refers to here. Is it businesses with big capital, businesses in several states, what is exactly being meant when one says 'they belonged in big businesses?"

**Response:** We have made changes on page 11, line 255 to indicate in bracket that big business means “business with large capital”.

4. References need to be added in some parts where sentences are used loosely. For example page 6, line 139 will benefit if a reference is added to show where the information is coming from.

**Response:** Reference to the statement has been added on page 6, line 140.

5. It is unclear what authors are referring to when on page 6. line 143-144 they say .."We conducted document review to collect the unit cost of different malaria services." What documents are being referred to here? Government documents? Peer reviewed documents that are publicly available and accessible? Clarity would help readers understand what type of documents are being referred to here.

**Response:** We have provided information on the documents that we reviewed. The documents were mentioned in track changes on page 6, line 144 to 145.

6. Page 11 line 251-253, I do not think the sentence indicating that the survey sample were evenly distributed among the five quintiles of the NDHS 2018 adds value since the whole point is to have these groups evenly distributed, unless I am missing something here or the claim being made is incomplete. Please, check.

**Response:** The statement has been deleted for clarity. This can be found on page 11, line 258.

7. There is interesting result showing that rural households paid more than twice OOP compared to urban households ($098 vs. $0.42 - See Table 2 on page 13). There is no discussion of why this is the case. Most of this is also shown to be on medical costs, why is this being the case? Self treatment and purchase of medicines at drug stores? more discussion on possible reasons for this spending by rural poor households would surely be warranted here.

**Response:** This important observation by the reviewer has been addressed in the discussion section, page 17, line 364 to 368 with references to corroborate the findings.

8. A definition of what medical costs includes would also be useful. Does this include drugs? consultation fees, bed net purchase, RDT Kits? laboratory tests? It is unclear since these are also listed separately in some instances.

**Response:** The definition of what is included in the medical costs have been highlighted on page 8, line 190 to 194

9. These findings in some way corroborates with other finding published from Sub Saharan Africa. For instance Njau JD et al (2013) "Exploring the impact of targeted distribution of free bed-nets on household bed-net ownership, socioeconomic disparities and childhood malaria infection rates: Analysis of National Malaria Survey data from three sub Saharan African Countries" found that donor funded distribution of bed-nets to targeted households benefited wealthier households than poorer households in Tanzania and Uganda, but not in Angola. All three countries were funded by the President Malaria Initiative (PMI). It would be nice for authors to review the paper and see what similarities they can draw from the paper in their discussions section.

**Response:** Thank you for sharing relevant work. We have included statement in track changes on page 16, line 351 to 355 to substantiate findings on malaria funded programmes in other African countries with reference.

10. The authors should also mention the limitation of Asset index in their study limitation section. The Asset Index is known to be a static wealth measure and is not a good reflection of households struggling for cash to pay for emergency malaria treatment service needs. For this reason it is important for readers to know the mechanics of Wealth Index measure and what it aims to achieve in these types of studies.

**Response:** We have now properly discussed the limitations of this studyon page 19, line 407 to 413.

11. I also think they should mention the fact that the study does not include the use or uptake of Intermittent Preventive Treatment of Malaria in pregnant women which is one of the core components of PMI funding. IPTp is an important malaria control strategy funded through PMI and the fact that the benefits of this strategy are not being reported here is a limitation that is worth discussing at least briefly.

**Response:** This has also been included in the Limitation section, page 19, 407 to 413.
